# A De Novo Deleterious *PHEX* Variant Without Clinical Features of X-Linked Hypophosphatemia

**DOI:** 10.1210/jcemcr/luad082

**Published:** 2023-10-19

**Authors:** Michelle Kayser, Preti Jain, Allen Bale, Thomas O Carpenter

**Affiliations:** Department of Pediatrics (Endocrinology), Yale University School of Medicine, New Haven, CT 06514, USA; Department of Genetics, Yale University School of Medicine, New Haven, CT 06514, USA; Department of Genetics, Yale University School of Medicine, New Haven, CT 06514, USA; Department of Pediatrics (Endocrinology), Yale University School of Medicine, New Haven, CT 06514, USA

**Keywords:** X-linked hypophosphatemia, skewed X-inactivation

## Abstract

X-linked hypophosphatemia (XLH), the most common form of hereditary rickets, is due to inactivation of *PHEX,* resulting in increased circulating fibroblast growth factor 23. Consequent renal phosphate loss leads to hypophosphatemia, rickets, and progressive bow deformity. Inheritance is X-linked dominant, such that heterozygous females are affected, as well as hemizygous males. A 10-month-old girl was referred for potential treatment for presumed XLH. Amniocentesis, performed following prenatal identification of duodenal atresia, polyhydramnios, and intrauterine growth restriction, revealed a *de novo* X-chromosomal deletion encompassing 10 genes, including *PHEX*. *Postnatal genetic testing confirmed presence of the deletion in the baby.* She demonstrated no phenotypic, biochemical, or radiographic features of XLH. Neither parent had features of XLH, nor carried the deletion. Given the discordance between genotype and phenotype, evaluation for skewed X-inactivation was pursued. Methylation analysis via the androgen receptor locus was inconclusive, thus RNA sequencing was pursued. Analysis of 12 high-quality single nucleotide polymorphisms (SNPs) that are expressed in mRNA revealed skewed X-inactivation. Heterozygous disruption of *PHEX* typically confers a diagnosis of XLH. Skewed X-inactivation, whereby one X chromosome is preferentially silenced, appears to have protected this patient from the expected expression of an X-linked dominant disorder.

## Introduction

X-linked hypophosphatemia (XLH) is the most common form of hereditary rickets; however, it is relatively rare with an estimated prevalence of 1 per 20 000 live births. It is inherited in X-linked dominant fashion, which is uncommon among Mendelian disorders. Damaging variants of *PHEX* (phosphate-regulating protein with homology to endopeptidases on the X chromosome), located at Xp22.1, cause XLH. *PHEX* is expressed predominantly in bone and teeth, and its inactivation results in increased circulating levels of fibroblast growth factor 23 (FGF23), which inhibit renal tubular phosphate reabsorption. The subsequent hypophosphatemia leads to bone demineralization and rickets. Major clinical findings in children with XLH include rickets, osteomalacia, growth failure, and dental abnormalities. Rickets is not evident at birth and takes several months to develop, so that early prenatal diagnosis does not appear to confer a prognostic advantage for this aspect of the disease. Noninvasive methods of prenatal genetic testing are becoming more sophisticated and expansive, leading to early detection of unanticipated genetic diagnoses. Here we present a unique case of an infant female who presented with a prenatally discovered chromosomal deletion encompassing *PHEX* without clinical, biochemical, or radiographic evidence of XLH, which was found to be the result of skewed X-inactivation.

## Case Presentation

**Figure 1.**
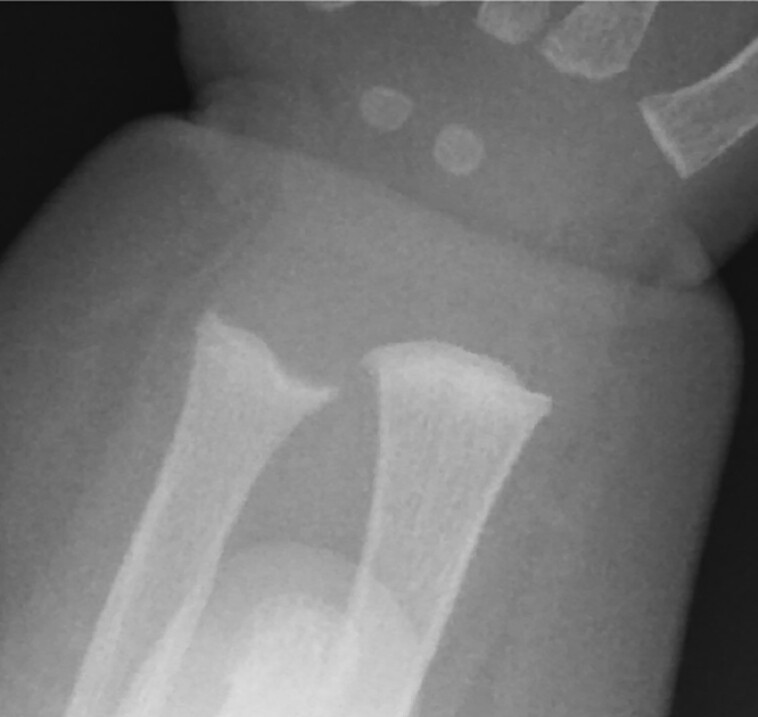
Radiograph of the left wrist, age 8.5 months.

**Figure 2.**
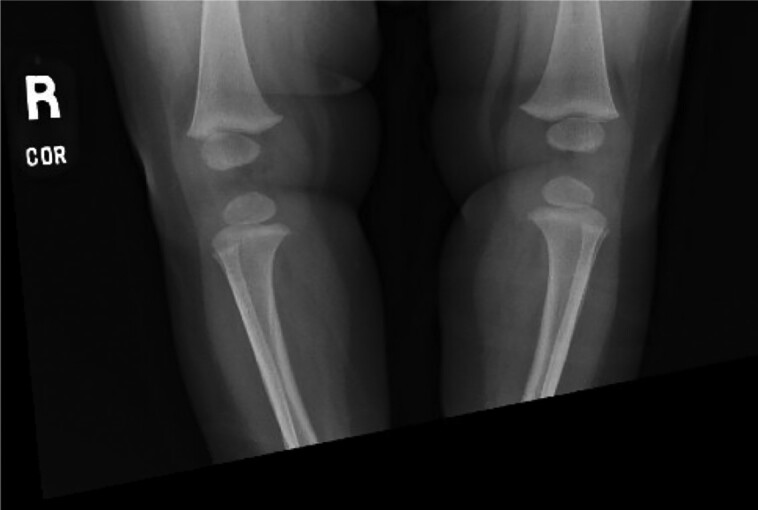
Radiograph of the bilateral lower extremities, age 8.5 months.

The patient was seen at our institution at 10 months of age in consultation regarding potential treatment with burosumab for presumed XLH. She was born at 37 weeks and 5 days gestation via urgent cesarean delivery for failure to progress, to a 36-year-old gravida 3 para 0 mother. Pregnancy was complicated by duodenal atresia, a 2-vessel umbilical cord, polyhydramnios, and intrauterine growth restriction. As duodenal atresia and 2-vessel cord are associated with trisomy syndromes, prenatal genetic testing was pursued. Amniocentesis revealed a ∼2-Mb X-chromosomal deletion encompassing 10 genes, including *PHEX* (Xp22.12 → P22.11: *MBTPS2, SMS, PHEX, CBLL2, DDX53, PTCHD1, PRDX4, ACOT9, SAT1, APOO*). Of these, *PHEX, PTCHD1, MBTPS2,* and *SMS* are associated with pathologic findings. Postnatal testing confirmed this X chromosome deletion in the infant; however, it was not present in either parent, confirming the de novo nature of the deletion. Postnatally she underwent uncomplicated repair of duodenal atresia at 5 days of age and was discharged home at 1 month of age. Seven teeth were evident by 11 months of age, and she met developmental milestones at appropriate ages. She exhibited normal linear growth, tracking between the 47th and 91st percentile. Family history was noncontributory, with no history of frequent fractures, abnormal dentition, or poor growth. Serial biochemical and radiographic studies obtained in the first year of life did not support a diagnosis of XLH (Table 1, Figs. 1 and 2). A mild elevation in c-terminal FGF23 was noted but appropriate in the context of a high-normal serum phosphorus level. Radiographic examination of the wrists revealed a mildly exaggerated contour of the ulnar metaphyseal area, but no fraying, cupping, or other features typical of active rickets. Radiographs of the knees were normal.

**Table 1.** Laboratory values for the patient

Blood Test	Normal Range	2 days	1 week	2 weeks	3 weeks	4 months	5 months	8 months	16 months	31 months
Sodium	136-144 mEq/L (136-144 mmol/L)	138 (138)	138 (138)	138 (138)	136 (136)	138 (138)	140 (140)	139 (139)		
Potassium	3.4-5.6 mEq/L (3.4-5.6 mmol/L)	4.1 (4.1)	4.2 (4.2)	4.5 (4.5)	4.4 (4.4)	5.7 (5.7)	4.5 (4.5)	4.3 (4.3)		
Chloride	95-108 mEq/L (95-108 mmol/L)	102 (102)	103 (103)	108 (108)	106 (106)	106 (106)	104 (104)	104 (104)		
CO2	20-30 mEq/L (20-30 mmol/L)	28 (28)	26 (26)	24 (24)	25 (25)	21 (21)	22 (22)	20 (20)		
BUN	2-20 mg/dL (0.71-7.14 mmol/L)	14 (5)	11 (3.9)	9 (3.2)	11 (3.9)	10 (3.6)	8 (2.8)	8 (2.8)		
Creatinine	0.3-0.7 mg/dL (0.02-0.06 mmol/L)	0.3 (0.02)	0.2 (0.01)	0.2 (0.01)	0.2 (0.01)	0.2 (0.01)	0.19 (0.01)	0.24 (0.02)		
Calcium	8.8-10.8 mg/dL (2.2-2.69 mmol/L)	10.3 (2.6)	10.9 (2.7)	10.3 (2.6)	10.2 (2.5)	11.2 (2.8)	10.3 (2.6)	10.5 (2.6)		10.0 (2.5)
Albumin	3.4-5.2 g/dL (0.51-0.78 mmol/L)	3.6 (0.54)	3.4 (0.51)	2.7 (0.40)	3.0 (0.45)	4.4 (0.66)	4.0 (0.60)	4.6 (0.69)		
Alkaline Phosphatase	150-420 U/L	90	134	135	186	203	246	238	270	232
Phosphorus	3.8-6.5 mg/dL (1.22-2.09 mmol/L)	5.3 (1.7)	6.2 (2.0)	6.4 (2.1)	6.1 (2.0)	7.4 (2.4)	6.5 (2.1)	5.9 (1.9)	5.2 (1.7)	4.2 (1.4)
Magnesium	1.5-2.5 mg/dL (0.62-1.03 mmol/L)	1.9 (0.78)	2.2 (0.90)	2.1 (0.86)	2.0 (0.82)					
Vitamin D 1,25	15-90 pg/mL (37.4-224.6 pmol/L)					145 (348)	67 (161)	114 (274)		64.3 (154)
Vitamin D,25	30-100 ng/mL (30-250 nmol/L)					182.8 (H) (456)	70.8 (177)	39.5 (98)		49.3 (123)
Intact PTH	10-65 pg/mL (1.06-6.89 pmol/L)					6.4 (0.67)	22 (2.33)	15 (1.59)		
FGF-23	44-215 RU/mL						223	279		

## Diagnostic Assessment

**Figure 3.**
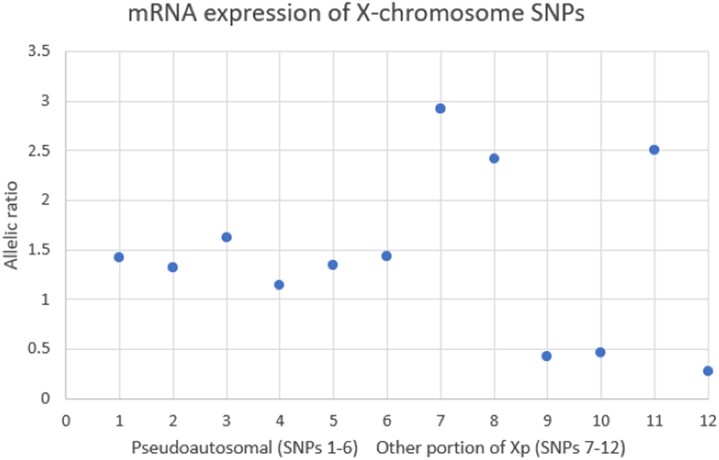
Plot of allelic ratios of SNPs from pseudoautosomal region and non-pseudoautosomal region. Pseudoautosomal SNPs maintain relative 1:1 ratios, non-pseudoautosomal SNPs show substantial deviation away from 1:1 ratio. *SNP*, Single nucleotide polymorphism.

The discordance between this patient's genotype and phenotype raised the consideration of skewed X chromosome inactivation. We investigated this possibility via a commercially available methylation analysis of the androgen receptor gene (*AR*) locus. Approximately 80% of females are heterozygous at the highly polymorphic CAG repeat in exon 1 of *AR*, allowing for differentiation between maternally and paternally inherited X chromosomes. A distinction between active and inactive X chromosomes can be determined by methylation analysis of enzyme restriction sites neighboring the *AR* locus, as methylation is evident on inactive X chromosomes, but not on active X chromosomes. Our patient was found to be homozygous for *AR*, and thus this testing was uninformative, a situation arising in 10% to 20% of cases. We thus sought an alternative means of examining the possibility of skewed X chromosome inactivation using RNA sequencing. We isolated mRNA from a fresh patient sample and converted it to cDNA, which was sequenced and mapped to the reference human genome hg19. Heterozygous, single nucleotide polymorphisms (SNPs) in coding regions were identified. In the absence of skewed X-inactivation, both alleles would be expressed equally, and the expected ratio between the alleles in RNA would be 1:1. Pseudoautosomal regions of the X chromosome escape X-inactivation, and a 1:1 allelic ratio for expression of SNPs in these regions is expected. A total of 12 high-quality SNPs were identified in our patient (6 non-pseudoautosomal, 6 pseudoautosomal) and expression patterns analyzed. In the pseudoautosomal regions, none of the allelic ratios differed significantly from 1:1. Among the 6 non-pseudoautosomal SNPs there was striking skewing from a 1:1 ratio. (Table 2, Fig. 3). Because parent of origin was not determined, the direction of skewing (excess expression of paternal vs maternal alleles) could not be determined. Data are presented conservatively as major allele vs minor allele without respect to parent of origin.

**Table 2.** A survey of SNPs on the p arm of the X chromosome

Gene	Chromosomal location	Allelic ratio	CI (confidence interval) (5%, 95%)
**Xp Pseudoautosomal Region SNPs**			
GTPBP6	X:229542	1.42	0.94, 2.22
PPP2R3B	X:322257	1.32	0.72, 2.54
CSF2RA	X:1428421	1.62	0.99, 2.67
SLC25A6	X:1508324	1.14	0.89, 1.48
ASMTL	X:1531648	1.35	0.78, 2.43
ZBED1	X:2408200	1.43	0.72, 3.12
**Xp Non-Pseudoautosomal Region SNPs**			
PRKX	X:3631167	2.92	1.65, 6.53
LOC389906	X:3735747	2.42	1.3, 5.64
CA5B	X:15800751	0.43	0.14, 1.19
ZRSR2	X:15838366	0.46	0.14, 1.30
TXLNG	X:16859628	2.50	1.19, 7.7
TIMP1	X:47444985	0.27	0.05, 0.96

## Treatment

This patient received no therapy for X-linked hypophosphatemia in view of the absence of clinical features of the disease.

## Outcome and Follow-up

At age 4 years, the time of our most recent contact, the patient has continued to exhibit normal growth and has not shown any phenotypic features consistent with XLH. Biochemical and radiographic studies have not demonstrated evidence of disease. Gene deletions of *PTCHD1*, *MBTPS2*, and *SMS* are associated with X-linked recessive disorders, such that carrier individuals are not affected*. MBTPS2* variants are associated with X-linked recessive genodermatoses, which encompass a variety of skin abnormalities (ichthyosis follicularis, atrichia, ectodermal dysplasia). Carrier females may have a normal phenotype or be mildly affected. Our patient has not exhibited any features of this gene deletion. Females carrying heterozygous *PTCHD1* and/or *SMS* deletions are unaffected.

## Discussion

Heterozygous loss of the *PHEX* gene, identified in this patient via prenatal genetic testing, typically confers a diagnosis of XLH. Our patient remains without the expected clinical or phenotypic features of XLH due to skewed X chromosome inactivation in which the mutant chromosome was preferentially silenced, thus protecting her from the disease state. X chromosome inactivation is responsible for gene dosage compensation in females to prevent X-chromosomal genes from being expressed at twice the level as males. X-inactivation generally occurs at random during early embryogenesis when one copy of the X chromosome is silenced and hypermethylation occurs on this inactive chromosome. Once inactivated, the X chromosome remains silent through subsequent mitotic divisions. Skewed X-inactivation, with preferential silencing of either the paternally or maternally inherited X chromosome, results in a nonrandom pattern of X chromosome inactivation with considerable implications on expression of disease in heterozygous females. For our patient, we hypothesize that the large X chromosome deletion conferred poorer survival of the cell line in which the mutant X chromosome remained transcriptionally active. Thus, a higher predominance of cells with the wild-type X chromosome persisted which ultimately protected her from disease. Other X-linked disorders have been subject to phenotypic modifications due to skewed X chromosome inactivation [1-3]. Hou et al [2] describes a female patient with Wiscott-Aldrich syndrome (WAS), an X-linked recessive disorder in which heterozygous females are carriers and do not manifest symptoms of the disease. After confirmation of a heterozygous damaging variant in the *WAS* gene, skewed X-inactivation was identified with predominant inactivation of the wild-type X chromosome. Thus, in contrast to the expected carrier state of heterozygous females, overt disease was evident due to the predominant expression of the disease-associated variant. Investigation of skewed X-inactivation should be considered in female patients in whom there is discordance between genotype and phenotype.

## Learning Points

- X chromosome inactivation is responsible for gene dosage compensation in females to prevent X-chromosomal genes from being expressed at twice the level as males.
- Preferential inactivation of either the maternally or paternally derived X chromosome produces a nonrandom pattern of X chromosome expression; allelic expression of 80:20 or greater is highly suspicious for skewed X-inactivation.
- Skewed X-inactivation has significant impact on disease expression in heterozygous females.
- Preferential silencing of the mutant chromosome masks disease state in X-linked dominant diseases.

## Contributors

All authors made individual contributions to authorship. M.K. and T.C. were involved in the diagnosis and management of this patient and manuscript submission. P.J. and A.B. conducted RNA sequencing analysis and preparation of figures. All authors reviewed and approved the final draft.

## Data Availability

Original data generated and analyzed during this study are included in this published article.

## Abbreviations

FGF23: fibroblast growth factor 23
SNP: single nucleotide polymorphism
